# Human Occupancy as a Source of Indoor Airborne Bacteria

**DOI:** 10.1371/journal.pone.0034867

**Published:** 2012-04-18

**Authors:** Denina Hospodsky, Jing Qian, William W. Nazaroff, Naomichi Yamamoto, Kyle Bibby, Hamid Rismani-Yazdi, Jordan Peccia

**Affiliations:** 1 Department of Chemical and Environmental Engineering, Yale University, New Haven, Connecticut, United States of America; 2 Department of Civil and Environmental Engineering, University of California, Berkeley, California, United States of America; 3 Japan Society of the Promotion of Science, Ichiban-cho 8, Chiyoda-ku, Tokyo, Japan; Ohio State University, United States of America

## Abstract

Exposure to specific airborne bacteria indoors is linked to infectious and noninfectious adverse health outcomes. However, the sources and origins of bacteria suspended in indoor air are not well understood. This study presents evidence for elevated concentrations of indoor airborne bacteria due to human occupancy, and investigates the sources of these bacteria. Samples were collected in a university classroom while occupied and when vacant. The total particle mass concentration, bacterial genome concentration, and bacterial phylogenetic populations were characterized in indoor, outdoor, and ventilation duct supply air, as well as in the dust of ventilation system filters and in floor dust. Occupancy increased the total aerosol mass and bacterial genome concentration in indoor air PM_10_ and PM_2.5_ size fractions, with an increase of nearly two orders of magnitude in airborne bacterial genome concentration in PM_10_. On a per mass basis, floor dust was enriched in bacterial genomes compared to airborne particles. Quantitative comparisons between bacterial populations in indoor air and potential sources suggest that resuspended floor dust is an important contributor to bacterial aerosol populations during occupancy. Experiments that controlled for resuspension from the floor implies that direct human shedding may also significantly impact the concentration of indoor airborne particles. The high content of bacteria specific to the skin, nostrils, and hair of humans found in indoor air and in floor dust indicates that floors are an important reservoir of human-associated bacteria, and that the direct particle shedding of desquamated skin cells and their subsequent resuspension strongly influenced the airborne bacteria population structure in this human-occupied environment. Inhalation exposure to microbes shed by other current or previous human occupants may occur in communal indoor environments.

## Introduction

Airborne bacteria in the indoor environment are the confirmed or presumed causative agents of several infectious diseases, and their components are linked to the development and exacerbation of chronic respiratory illness including asthma [1], [2], [3], [4], [5], [6]. These associations are important in industrialized countries and in cities of emerging nations where people spend at least 85% of their time indoors [7], [8], [9]. Developing a fundamental understanding of the origins and character of biological aerosols is therefore a research priority for reducing human exposure to airborne pathogens and bacterial toxins in the indoor environment [10].

Studies based on indoor/outdoor mass balance and receptor-based source apportionment models have demonstrated that, in addition to particles suspended in outdoor air, material resuspended from surfaces as a result of human activities is an important source of indoor airborne particles [11], [12], [13]. Other significant sources of indoor airborne bacteria may be human oral and respiratory fluid emitted via coughing, sneezing, talking, and breathing [14], [15], [16] or the direct shedding of skin-associated microbiota [17], [18], [19]. Ribosomal rRNA sequences that are homologous to the sequences of bacteria commonly present on human skin have been found in indoor floor dust [20] suggesting that resuspension of this dust may also act as a human-associated source of airborne bacteria or bacterial constituents. Previous studies have extended characterization of these potential sources to indoor air content by estimating occupancy-associated emission rates of culturable bacteria [21], and — through the use of biomarker analysis — tracking the sources of some bacteria isolated from indoor air back to human origin [19], [22]. However, to date, there are no reports in the literature that directly compare phylogenetically derived indoor air bacterial populations with populations from potential sources including human occupants, ventilation duct air and floor dust.

The purpose of the research reported in this paper is to investigate the sources and origins of bacteria in indoor air in a university classroom. We hypothesize that through resuspension and direct shedding, human occupancy strongly influences the concentration and character of bacteria in indoor air. To test this hypothesis, quantitative measurements of airborne particle mass and bacteria concentrations were performed in an instrumented classroom during occupied and vacant conditions. To determine the contributions of other sources and to elucidate the origin (human or environmental) of bacteria suspended in indoor air, phylogenetic libraries were produced for indoor aerosols during occupancy and for potential indoor aerosol sources that include floor dust, ventilation duct air entering the room, and particles collected on the building's HVAC filter. The microbial ecology results were further compared to published phylogenetic libraries of the human skin microbiome, outdoor bioaerosols, and indoor floor dust to help assess the relative abundances of source-associated bacteria found in indoor air and to determine if the trends observed in the classroom studied herein would be more generally applicable. This work integrates knowledge of physical indoor aerosol processes with molecular biology-based tools to determine the origins of bacteria in indoor air and complements a recent study that reports the size-distributed emission rates of airborne bacterial populations in classrooms owing to human occupancy [23]. Study results provide insight into how humans are exposed to indoor microorganisms originating from the environment and other humans. Such insight can help inform how buildings might be designed, operated, and occupied to reduce human exposure to bacteria that cause adverse health effects.

## Results

### Room Conditions and Ventilation Configuration

Experiments were conducted in a small university classroom during four days under vacant conditions and during three additional days under occupied conditions. Continuously monitored environmental parameters included temperature, relative humidity (RH), and CO_2_ concentrations. For the seven sampling days, the outdoor temperature and relative humidity (mean ± standard error) during the time of sampling were 13.4±0.9°C and 45±6.0%, respectively. Corresponding indoor temperature and relative humidity were 23.5±0.4°C and 28±2.6%. The measured outdoor CO_2_ concentration was consistent with the tropospheric background concentration (390 ppm) and the indoor difference between occupied and vacant periods averaged 230 ppm.

Investigation into the building air handling system revealed that the ventilation duct air that supplied the room was a mixture of outdoor air and building return air from other classrooms and offices in the building. The proportion of outdoor air to total air flow varies from 25% to 100% depending on the building's heating and cooling needs, and would have been near 50% during the experiments conducted herein based on the outdoor temperature. Before entering the classroom, the air mixture passes through a HVAC filter with a MERV 8 rating. The efficient removal by this filter of airborne particles >3 µm was confirmed through optical counter measurements (Figure S1). Thus, the HVAC filter dust results presented herein represent a cumulative sample of airborne particulate matter dominated by the coarse fraction of outdoor air and indoor return air particles collected over the filter operation period of 6 months from August to February. The ventilation duct supply sample represents mainly the smaller particles that pass through the filter and enter the study room.

### Aerosol Measurements

**Figure 1:**
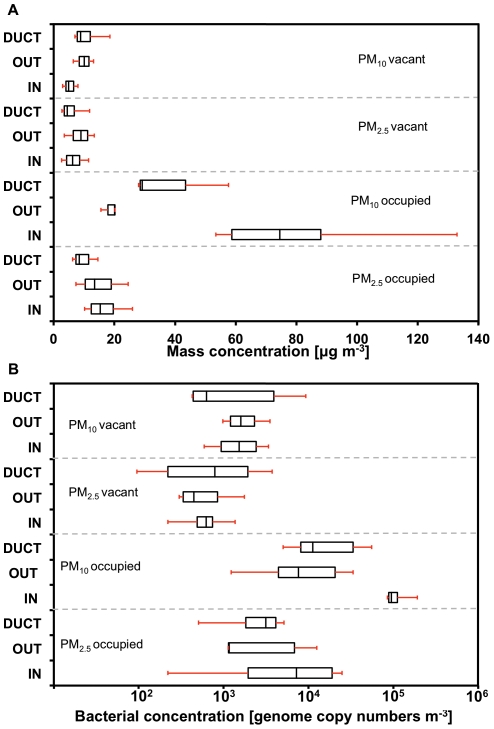
Airborne mass and bacterial genome concentrations. Box and whisker plots of (**A**) total particle mass and (**B**) bacterial genome copy number (GCN) measured in indoor air, ventilation duct supply air, and outside air during occupied and vacant periods. The box frames the upper quartile and lower quartile, the line represents the median, and whiskers denote range.

Aerosol samples in this study include indoor, outdoor, and ventilation duct supply PM_10_ (mass of particulate matter in particles 10 µm in aerodynamic diameter or less) and PM_2.5_ (mass of particulate matter in particles 2.5 µm in aerodynamic diameter or less) mass concentrations (Table 1). The samples were obtained during occupied and vacant periods to characterize the influence of occupancy on airborne particles and airborne bacteria. For phylogenetic comparisons, floor dust and HVAC filter dust from the HVAC system's filter (from hereon referred to as “HVAC filter dust”) were mechanically extracted and then sieved and resuspended to obtain PM_37_ (mass of particulate matter in particles 37 µm in aerodynamic diameter or less), PM_10_, and PM_2.5_ size fractions. A full description of samples collected and analyzed is presented in Table 1. Total mass and bacterial genome concentrations for the PM_10_ and PM_2.5_ size fractions in air are shown in Figure 1. Occupancy results in an increase in airborne concentrations of both total particle mass and bacterial genome copy numbers (GCN). For indoor PM_10_, mass increased by 15 times (*p* = 0.00001) and GCN increased by 66 times (*p* = 0.001) for occupied conditions compared with the vacant case, while smaller increases of 2.5 times (*p* = 0.015) and 16 times (*p* = 0.02) occurred in PM_2.5_ mass and GCN, respectively. Ratios ± standard error of PM_10_ to PM_2.5_ mass concentrations were 4.9±0.3 for indoor occupied air, 0.8±1.2 for indoor unoccupied air, 1.2±0.9 for occupied outdoor air and 1.1±0.9 for vacant outdoor air, respectively, indicating a strong influence on respirable particles larger than 2.5 µm for the indoor environment when occupied, and substantiating the expectation that occupancy is an important contributor to suspended coarse particulate matter [24].

**Table 1:** Airborne particulate matter, filter dust, and floor dust samples acquired and analyzed in this study.

Sample category	Sample description	Processing	No. collected	No. used in mass analyses	No. used in qPCR analysis	No. used in sequencing
Indoor air	Indoor air, occupied, PM_10_	Sampled onto PCTE filters	6	6	6	5
	Indoor air, occupied, PM_2.5_	Sampled onto PCTE filters	6	6	6	_
	Indoor air, vacant, PM_10_	Sampled onto PCTE filters	8	8	8	_
	Indoor air, vacant, PM_2.5_	Sampled onto PCTE filters	8	8	8	_
Ventilation duct supply air	Ventilation duct supply air, occupied, PM_10_	Sampled onto PCTE filters	3	3	3	4 (3 samples, one sequencing duplicate)
	Ventilation duct supply air, occupied, PM_2.5_	Sampled onto PCTE filters	3	3	3	_
	Ventilation duct supply air, vacant, PM_10_	Sampled onto PCTE filters	4	4	4	_
	Ventilation duct supply air, vacant, PM_2.5_	Sampled onto PCTE filters	4	4	4	_
Outdoor air	Outdoor air, occupied, PM_10_	Sampled onto PCTE filters	3	3	3	_
	Outdoor air, occupied, PM_2.5_	Sampled onto PCTE filters	3	3	3	_
	Outdoor air, vacant, PM_10_	Sampled onto PCTE filters	4	4	4	_
	Outdoor air, vacant, PM_2.5_	Sampled onto PCTE filters	4	4	4	_
Floor dust	PM_37_	Sieved	12	−	−	3
	PM_10_	Sieved, resuspended, and sampled on PCTE filters	12	12	12	1
	PM_2.5_	Sieved, resuspended, and sampled on PCTE filters	12	12	12	_
HVAC filter dust	PM_37_	Sieved	4	_	_	_
	PM_10_	Sieved, resuspended, and sampled on PCTE filters	4	_	_	3

**Figure 2:**
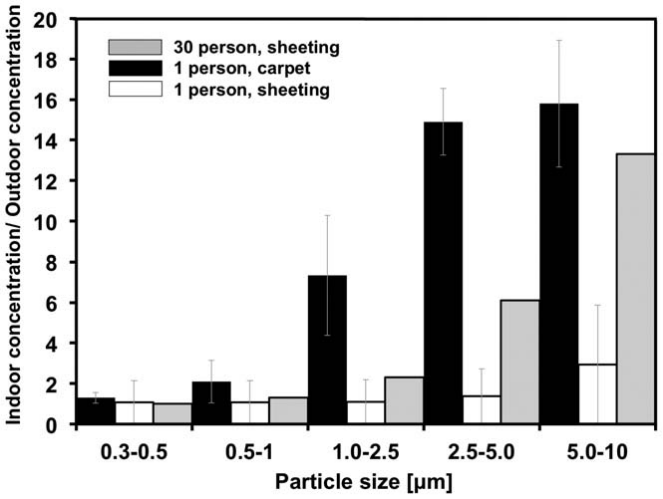
The influence of floor dust resuspension and particle shedding on particle number concentrations of varying optical diameter. Plotted are the ratio of occupied indoor to simultaneous outdoor particle number concentrations for five size ranges from 0.3 µm to 10 µm under the following three conditions. Black bars represent the case of 30 people sitting on a carpeted floor that is covered with plastic sheeting (to prevent resuspension of floor dust). White bars represent one person walking on a carpeted floor covered with plastic sheeting. Gray bars represent one person walking on a carpeted floor (without plastic sheeting). Error bars indicate one standard error of the mean for replicate experiments. The experiment in which 30 people were sitting on a carpeted floor covered with plastic sheeting was conducted only once.

To elucidate potential sources of increased aerosol concentrations during occupancy, experiments were conducted to investigate separately the impacts of resuspension from the carpet during walking and direct shedding from humans. The ratio of the indoor particle number concentration to the outdoor particle number concentration for three experimental conditions are presented in Figure 2. These conditions included (a) one person walking on the carpet, (b) one person walking on the same floor covered with plastic sheeting to eliminate resuspension of particles from the carpet, and (c) 30 adults occupying the room while the carpet was covered with plastic sheeting. For condition (c), occupants were allowed to moved freely about the room and activities centered on talking, reading, and writing. The results in Figure 2 suggest that significant particle generation in the occupied test room may occur through resuspension of dust deposited on the floor, through direct shedding of particles from human occupants, or both. Cases (a) and (c) resulted in particle number concentrations that were greater than the outdoor concentrations for all size ranges. In case (a) when the carpet was not covered with plastic sheeting (resuspension), these increases were 1.2 to 11 times across the range of particle sizes with an average increase of 5.2 times (*p* = 0.05). In case (c), proportional increases of 1.2–4.5 times with an average of 2.7 times (*p* = 0.18) were observed for the floor covered with plastic when the occupancy level was 30 people. Case (c) is suggestive of shedding rather than resuspension. In both cases, the proportional extent of particle concentration increase rose monotonically with increasing optical particle size throughout the instrument's measurement range.

**Figure 3:**
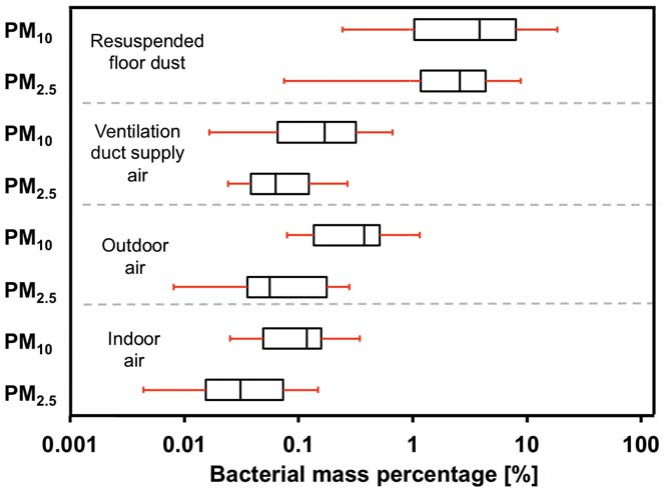
Enrichment of bacteria in airborne particulate matter and floor dust. Bacterial mass percentage (100×bacterial mass divided by total particle mass) in indoor air, outdoor air, and duct supply air samples and in the PM_2.5_ and PM_10_ size fraction of resuspended floor dust samples. Mass fractions were estimated assuming an average mass of 655 fg per bacterium [25]. Box and whisker plots have the same interpretation as in Figure 1.

A comparison of the bacterial mass percentage of airborne particle and floor dust samples is shown in Figure 3. Estimates of bacterial mass were computed assuming the mass of a bacterium to be 655 femtograms [25], and an average 16 S rDNA gene copy number of four per bacterium [26]. Results displayed in Figure 3 demonstrate that the PM_10_ and PM_2.5_ fractions of resuspended floor dust are enriched with bacteria, compared to indoor air, ventilation duct supply air, and outdoor air. The median bacterial mass percentages of indoor and outdoor airborne particles were less than 0.3%, whereas the bacterial proportion of aerosolized floor dust exceeded 2.2% in both size fractions. Based on a Tukey's range test, resulting ranks for bacterial abundance in both PM_2.5_ and PM_10_ cases are resuspended floor dust≫outdoor air>duct supply air>indoor air. However, only differences between resuspended floor dust and the three air samples were statistically significant at a 95% confidence level.

### Phylogenetic Analysis

Sixteen samples from indoor air, ventilation duct supply air, floor dust, and HVAC filter dust (Table 1) were analyzed for bacterial population composition using the 454 GS-FLX pyrosequencing platform with multiplex identifiers (MIDs). Sample MIDs are presented in Table S1. After machine- and method-based quality control, denoising, and chimera checking, 10,675 partial 16 S rDNA gene sequences were generated at an average trimmed length of 500 base pairs (bp). Rarefaction values based on 97% similarity were produced for each sample and were then averaged based on their sample type (Figure S2). Rarefaction curves of observed OTUs continued to rise with increasing numbers of sequences, suggesting that further increases in sample size would yield more species. Chao1 richness estimator predicted 3720, 1260, 2990, and 640 OTUs, respectively, for floor dust, HVAC filter dust, indoor air, and ventilation duct supply air (Figure S1). The diversity metrics reported here are higher than those previously determined for floor dust, which ranged from 83 to 464 based on the Chao1 approach in conjunction with a cloning and Sanger sequencing method [20], [27].

**Figure 4:**
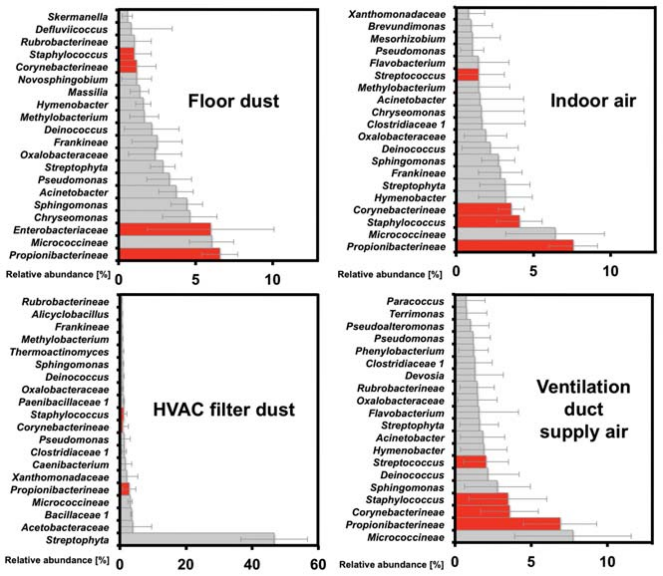
Relative abundances of bacteria in the indoor air, ventilation duct air, floor dust, and HVAC filter dust samples. Relative abundances of the 20 most common bacterial taxa in indoor air, ventilation duct air, HVAC filter dust, and floor dust. Indoor and ventilation duct air include PM_10_ samples from indoor air when the room was occupied. Floor dust samples were sieved PM_37_ floor dust and resuspended PM_10_ floor dust taken after occupancy. HVAC filter dust represents samples from the filter of the building HVAC system that handled a variable mixture of outdoor air and indoor return air. Taxa are classified to the highest taxonomic level to which they could be confidently assigned. Error bars represent one standard error of the mean for nine indoor air PM_10_ samples, four floor dust samples, and three HVAC duct samples. Groups shown represent 55% of floor dust, 83% of HVAC filter dust, 51% of indoor air taxa, and 46% of ventilation duct air taxa.

The relative abundances of the 20 most prominent bacterial taxa from indoor air, ventilation duct supply air, HVAC filter dust, and floor dust are shown in Figure 4. (Phyla level data are presented in Figure S3.) Indoor air, ventilation duct supply air, and floor dust samples show heavy representation from the dominant bacteria previously found to be associated with human skin, hair, and nostrils [28], [29], [30], [31], [32]. These five human associated taxa — *Proprionibacterineae*, *Staphylococcus*, *Streptococcus*, *Enterobacteriaceae*, and *Corynebacterineae* — comprise 17%, 20%, and 17.5% of all bacteria in samples of indoor air, floor dust, and ventilation duct supply air, respectively. The HVAC filter dust sample demonstrated significant differences from all other samples, being strongly dominated by the *Streptophyta* phylum (chloroplast 16 S rRNA encoding gene from plants) with only minor (3%) representation from the five human-associated taxa described above.

**Figure 5:**
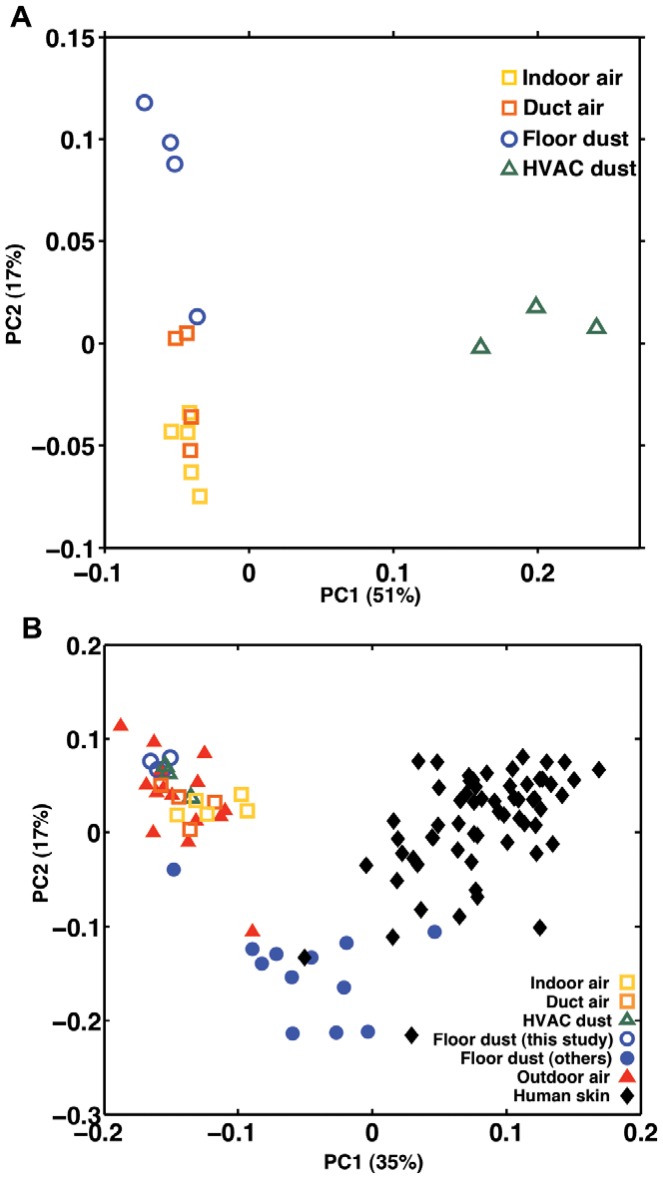
Comparison of indoor bacterial populations. (**A**) Weighted UniFrac-based bacterial diversity principal coordinate analysis of indoor air (yellow outlined squares), ventilation duct supply air (orange outlined squares), floor dust (outlined circles) samples, and HVAC filter dust samples (outlined triangles) from this study. (**B**) UniFrac-based bacterial diversity principal coordinate analysis displaying the two coordinates that explain most of the variation between samples from this study (open squares, circles, and triangles) and the bacterial ecology of human skin samples (filled diamonds) from Costello et al. [31], outdoor air samples (filled triangles) from Bowers et al. [33], and floor dust samples from Täubel et al. [20] and Rintala et al. [27] (filled circles).

To quantitatively compare populations, the similarities and differences between the sample bacterial community structures are presented in relation to principal coordinate analysis (see Figure 5A) on a weighted-UniFrac basis. Stemming from *p*-test significance evaluation using the Bonferroni correction for multiple comparisons, the bacterial communities characterized in indoor air and duct air during human occupancy were significantly different from the communities collected on the HVAC filter dust sample (*p*<0.001). Differences were not statistically significant between indoor air and ventilation supply duct air bacterial communities (*p*>0.1), or between ventilation supply duct air and floor dust communities (*p*>0.1). Indoor air bacterial communities reveal almost significant differences compared to those of floor dust (0.05<*p*<0.1).

Figure 5B displays the indoor air, ventilation supply duct air, HVAC filter dust, and floor dust samples in this study along with other samples from published studies on the microbial diversity of potential sources including floor dust, human skin, and outdoor air. In all, 104 samples were evaluated for this comparison: 16 samples from the present study; 12 floor dust samples from nursing homes and private residences [20], [27]; 15 outdoor air samples taken in areas with varying land use types including urban, rural, and agricultural sites [33]; and 61 human skin samples from two female and two male individuals sampled at different times including left and right palms, index fingers and forearms [31]. The weighted UniFrac analysis, which encompasses several different environments, demonstrates distinct groupings for aerosol samples, for human skin samples, and for floor dusts samples. The data show broad similarities among outdoor and indoor aerosol bacterial ecology, likely owing to the presence of many environmentally associated organisms in the indoor air samples taken in this study (Figure 4). Larger differences are observed in floor dust samples across studies, with the floor dust measured here (open blue circles) residing more closely to aerosol samples, and floor dust from nursing homes and private residences (closed blue circles) [20], [27] clustering more closely to human skin samples than to aerosol samples.

## Discussion

This study advances knowledge about the sources, origins, and character of bacterial aerosols in indoor settings through two main findings. First, human occupancy produces a marked concentration increase of respirable particulate matter and bacterial genomes. Second, bacteria from human skin and from other environmental sources significantly contribute to indoor air bacterial populations.

### Biological and PM_10_ aerosol concentrations during occupancy

Box and whisker plots of bacterial GCN and total particulate matter concentrations for occupied and vacant cases (Figure 1A–B) demonstrate that human occupancy produces a 15× increase in PM_10_ mass and a 66× increase in PM_10_ airborne bacterial genomes when compared to the vacant room case. These numbers relate to a PM_10_ increase of 75 µg m^−3^ during occupancy, compared to the average background outdoor concentration of 15 µg m^−3^, and an addition of 65,000 GCN m^−3^ compared to an average background outdoor concentration of 4,600 GCN m^−3^. Analogous increases, but with smaller proportionality factors, were also observed in PM_2.5_ fractions. Here, the increase during occupancy represented 10 µg m^−3^ (against a 12 µg m^−3^ average outdoor background) and 5,600 GCN m^−3^ (against a 1,600 GCN m^−3^ average outdoor background). These trends extend the findings from previous indoor particle resuspension studies, in which a strong direct dependence of resuspension rate on the size of abiotic particles has been reported [24]. Our findings also reinforce and extend prior observations about the contribution of occupancy to increases in coarse-particle bacterial marker concentrations and the association of bacteria with coarse particles emitted from desquamated human skin [18], [22]. The increases in airborne total particle mass concentrations during occupancy, above both indoor vacant and outdoor airborne concentrations, are also consistent with a broad range of particulate matter studies in diverse indoor environments. These studies suggest that, in the absence of smoking or cooking, resuspension is a dominant source of airborne particulate matter in occupied indoor environments [34], [35], [36].

Three additional lines of evidence reinforce the importance of resuspension in shaping the bacterial populations suspended in indoor air. First, bacterial mass per mass of particles was enriched in floor dust by an order of magnitude (Figure 3) compared to the bacterial mass percentage in particles collected from indoor air, from outdoor air, or from the ventilation duct supply air. Thus, emissions from this floor represented an enriched source of suspended bacteria per particle mass. Such enrichment also supports our observation that occupancy generates a 66× increase in bacterial GCN in indoor air, greater than the 12× increase in total airborne particle mass. A second line of evidence supporting floor dust as a source of airborne bacteria derives from quantitative comparisons of bacterial population structure in indoor air with potential sources (Figure 5A): the floor dust bacterial population in the test environment was similar to the ecology of indoor air. Finally, ventilation duct air that supplied the room showed higher concentrations of PM_10_ total mass and bacterial GCN during occupancy than during vacant periods. Such increases indicate that activity throughout the building during human occupancy results in a greater concentration of particulate matter and bacterial aerosols in other rooms and increased concentrations in the return air component of the ventilation system that supplied the study room.

### Human and environmental origins of bacteria suspended in indoor air

While the qPCR data demonstrate an increase of airborne bacteria due to occupancy and the principal coordinate population-based comparisons point to the importance of the resuspension of floor dust, neither approach fully elucidates the fundamental origin of bacteria in indoor air. Insight into the origin of indoor air bacteria can be gained by considering the most abundant taxa contained in the potential sources. For indoor air, ventilation duct supply air, floor dust, and HVAC filter dust, 17%, 17.5%, 20%, and 3% respectively, of the total bacterial abundance was comprised of human associated taxa — *Propionibacterineae*, *Staphylococcus*, *Streptococcus*, *Enterobacteriaceae*, and *Corynebacterineae* [28], [29], [30], [31], [32], [37]. Unique indicators of the human oral cavity and saliva, including *Fusobacterium* and *Veillonella* [31], [38], [39], [40], [41], [42], were also found in the indoor air and floor dust samples, although at very low abundances of 0.02% and 0.1%, respectively. These oral cavity and saliva-associated taxa were neither found in the ventilation duct supply air nor in the HVAC filter dust samples. The evidence suggests that, although emissions from the oral cavity are present, they were less important contributors to overall indoor airborne bacterial loads than emissions from human skin. Other recent investigations of floor dust have demonstrated the presence of skin-associated taxa [20], [27]. The present study leverages new methods in high-throughput DNA sequencing to extend these ecologies to indoor air as well as to identify potential sources of human-associated bacteria including floor dust and ventilation duct supply air.

Results from previous studies and the data collected here demonstrate that, during occupancy, resuspension and direct shedding of microorganisms from humans are potential sources of bacterial aerosol particles. The origin of many of the airborne bacteria is from human skin, hair, nostrils, and the oral cavity. It has been estimated that humans shed roughly a billion skin cells daily [43], with each square centimeter of skin per human hand having a concentration between 10^2^ to 10^7^ bacteria [44]. Desquamated human skin cells are an important contributor to particles in indoor air, and there is strong evidence that bacteria are associated with these skin cells [43]. Skin shedding may influence indoor air concentrations both through skin cells and their fragments directly becoming airborne, and also by deposition of cells onto floors and other surfaces followed by fragmentation and resuspension. Figure 2 demonstrates that room occupancy with the resuspension mechanisms inhibited (through the use of plastic sheeting on the floor) still yields particle number concentrations that are significantly greater than outdoor levels.

The indoor air, ventilation supply duct air, HVAC filter dust, and floor dust include taxa of environmental origin such as *Sphingomonadaceae*, *Rhodobacteraceae*, and *Streptophyta* (chloroplasts from land plants). Although environmental organisms are not as clearly defined as organisms of human origin, each of the three listed above have been previously reported in outdoor bioaerosol microbial ecology investigations [33], [45], [46], [47]. The presence of these environmental taxa in indoor air illustrates the potential importance of outdoor air particles conveyed through infiltration or ventilation and/or the tracked-in contribution of outdoor material to floor dust that is subsequently resuspended [12]. *Streptophyta* were found in high abundance in the HVAC filter dust samples (∼45%) and at lower abundance (2–4%) in the indoor air, ventilation duct supply air, and floor dust samples. The larger plant particles in outdoor air would be captured efficiently on the HVAC filter (capture efficiency is high for particle sizes larger than 3 µm, Figure S3), while individual bacteria may pass through the ventilation filter. The outdoor environment near the building was highly vegetated, being situated on a tree-lined street with maintained lawns and flower gardens, and there were no green plants in the room, nor were they common throughout the building. Sampling occurred in the fall during foliage change. Thus, decomposed material would be widely present outdoors. The finding of *Streptophyta* in indoor air and floor dust likely resulted from some combination of outdoor air infiltration into the building, tracked in dust from outdoors, or tracked in particles on the clothes, skin, and hair of people entering the building. *Streptophyta* abundance of the HVAC filter dust from this study is comparable with previously reported *Streptophyta* enrichments in alpine air (44%) [48], and urban air (19.9%) [33].

Finally, we note that this study was designed as an in-depth investigation of a single environment characterized by air-exchange rates, occupancy levels, and flooring types that are typical of buildings in industrialized countries. While this design allows for a more mechanistic understanding of the sources and origins of bacteria in indoor air, it does so with the limitation that variation among buildings (and across seasons) was not considered. Future investigations should extend this line of inquiry to multiple environments and should also consider variable occupancy levels and flooring types. Additional chamber-based studies that isolate humans during prescribed activities will be required to determine the range of emission rates and ecologies of directly shed bacteria. The limitation of only one environment sampled was partly ameliorated by considering external samples in the weighted UniFrac-based principal coordinate analysis in Figure 5B. Phylogenetic data from outdoor air in rural, agricultural, and urban settings and from floor dust in private residences and nursing home facilities were considered along with phylogenetic data from the human skin microbiome. The pooling of studies suggests that while bacterial ecologies present in indoor air or floor dust have consistent contributions of human- and environmentally associated bacteria, a significant amount of variability in these relative contributions occurs from building to building. These differences correspond to known relative abundances of human microbiota in floor dust. Specifically, the floor dust measured herein (open blue circles in Figure 5B) had only 15 to 20% content of human microbiome taxa and clustered with the aerosol samples, whereas the referenced floor dust studies from commercial buildings and private residences (closed blue circles) had >75% content of human taxa in each case [20], [27], and resided more closely to human skin samples than to aerosol samples. Overall, these data suggest (as one might have anticipated) that the relative contribution of human-associated bacteria is variable and environment-dependent. Characterizing the influence of design, operational and occupancy differences among buildings that account for these indoor air ecology differences is identified as an important future area of study.

### Conclusion

The integration of aerosol science with modern microbial ecology has revealed new insights into the sources and origins of airborne bacteria in indoor environments. Quantitative monitoring of indoor and outdoor air revealed that human occupancy is a dominant factor that contributes to the concentration of indoor airborne bacterial genomes. During occupancy, it appears that both resuspension from carpet and direct human shedding contributed to significantly elevate respirable particulate matter and bacterial concentrations above background concentrations. Similarities between indoor air populations and bacteria associated with the human skin microbiome point to the important contribution of human microflora. This work extends previous microbial ecology-based observations of human microflora from floor dust into indoor air, where exposure occurs. An important public health consequence of these results is that, through direct inhalation of resuspended or shed organisms, there is potential for current or previous occupants of a room to contribute substantially to inhalation exposure to bioaerosols.

## Methods

### Aerosol, floor, and HVAC sampling

The study site was a 90-m^3^ (L = 5.9 m, W = 4.9 m, H = 3.1 m) room whose floor was covered with lightly worn, commercial, medium pile, level-loop carpet. This classroom was located on the first floor of a five-story building on a university campus in the northeastern United States. One classroom wall bordered the outside of the building and the opposing wall contained a doorway opening to a hall. There was no visible water damage or known history of water damage in the building. The location adheres to a continuous cleaning schedule that includes vacuuming every second day and semiannual wet carpet cleaning. The room was mechanically ventilated and students and teachers were asked not to open windows and doors during the sampling campaign. Air movement followed a mixed ventilation configuration, with the ventilation supply air register located near the ceiling and outlets located on the floor at the wall opposite the supply air register. Outdoor samplers were located on the outside window ledge approximately 20 cm away from the window and 1.5 meters from the ground. Indoor air samplers were located near the middle of the room at a height of 1.5 m, and ventilation duct air samplers were placed in the supply duct air discharge register located above the room door. Based on carbon dioxide release and decay experiments measured with a LI-COR 820 CO_2_ analyzer (Licor Environmental, Lincoln, NE) the room air-exchange rate (AER) was determined to be 5.5±1.3 h^−1^ (mean ± standard deviation). Sampling was conducted on three occupied days, and on four vacant days during the fall of 2009. For the three occupied sampling days, the average human occupancy during the cumulative 22.2 hours of sampling was 4.7 persons. All necessary permits were obtained for the described field studies. Experiments were cleared through the university's environmental health and safety office and permission was granted from all classroom instructors.

Table 1 summarizes all particle and dust samples acquired during this study and provides information on the analysis for each sample type. Aerosol samples were collected in duplicate on each of the seven sampling days for both PM_10_ and PM_2.5_ in indoor air. One PM_10_ and one PM_2.5_ sampler each collected outdoor air and ventilation duct supply air. For each of the occupied days, samplers were started when occupancy occurred and then stopped one hour after occupancy ended, typically 5 to 8 hours. For each of the vacant days, the room was sampled for 8 hours during normal classroom hours. Indoor, ventilation duct supply air and outdoor sampler filters were collected and changed after each occupied and vacant day.” All PM samples were analyzed for mass and bacterial concentrations, and a subgroup of indoor air, ventilation duct supply air, and HVAC filter dust samples were used for phylogenetic library production in accordance with the schedule reported in Table 1. Each PM sample was collected on a 0.8-µm pore-sized, 37-mm diameter sterile polycarbonate track-etched (PCTE) filter that was loaded into commercial PM_10_ and PM_2.5_ samplers (Personal Exposure Monitors, SKC, Eighty Four, PA, USA) operated at 10±0.5 liters per minute. Occupied samplers were operated for a cumulative 22.2 hours during the three occupied sampling days, with sampling started at the onset of classes and stopped approximately one hour after the end of the last class each day. Unoccupied samplers were operated on weekends during typical classroom hours for approximately nine hours per day.

Floor dust was collected using a high-volume vacuum sampler (Eureka MightyMite Canister Vacuum, Eureka Company, Bloomington, IL, USA) fitted with a Mitest adapter and dust filter (Indoor Biotechnologies, Charlottesville, VA, USA) [48]. Floor dust samples were collected each day for occupied and vacant conditions and each sample was a composite of five randomly selected 20 cm×30 cm portions of flooring in commonly trafficked areas of the room. Prior to analysis, floor dust was processed by means of sieving to produce a 37 µm and smaller size fraction. Sieving results in a more homogenous mixture and selects for the smaller size range of dust that can potentially become aerosolized. A portion of this size fraction was also resuspended in a 0.66 m^3^ chamber and collected onto SKC Personal Exposure Monitoring PM_10_ and PM_2.5_ filters in accordance with the method described by Viau et al. [49].

Dust samples from the filter in the HVAC system that processed a blend of outdoor supply air and recirculated inside air were also collected; these samples are referred to as “HVAC filter dust” in this study. As described in the results section, the HVAC filter dust represents an aggregate sample of particles collected over the filter operation period of 6 months (August 2008 to February 2009). Representative portions of the filter material from the top, bottom, left, and right side of the used unit were removed and filter dust collected for subsequent processing and analysis. HVAC filters have previously been used as a sampling mechanism for indoor bioaerosols [49].

### PM_10_ and PM_2.5_ mass analysis

To determine particulate matter mass concentrations, filters were weighed before and after sampling. Weighing was performed using a precision balance (Mettler Toledo type XP6, Columbus, OH, USA). Static electricity was removed with a polonium α-particle source (Staticmaster static eliminator, NRD, Grand Island, NY, USA) and prior to weighing, filters were equilibrated at constant temperature and humidity (30°C±0.5°C, 31%±2% RH) for at least 24 hours.

### DNA extraction and quantitative PCR

The quantification of bacterial genomes from samples of indoor air, outdoor air, ventilation duct supply air, and resuspended floor dust was achieved using TaqMan real-time PCR. A three-stage DNA extraction method specifically developed for low concentration aerosol samples was utilized [50]. Briefly, cells on one half of the PCTE filter were lysed by enzymatic treatment and physical disruption through bead beating. Next, phenol/chloroform isoamyl alcohol extractions were used to isolate nucleic acids. Finally, DNA purification and concentration was conducted using spin columns and reagents from the Mobio PowerMax Soil DNA extraction kit (Mobio, Carlsbad, CA, USA). Exceptions to the cited method included proteinase K incubation at 54°C instead of 37°C, omitting the freeze-thawing cycle during DNA extraction, and omitting the 1-hour, 65°C incubation step prior to bead beating. The spin column was eluted two times in 100 µl of 10 µM Tris buffer (pH = 8). This sample was freeze-dried and re-eluted in 40 µl of 10 µM Tris buffer before amplification.

Quantitative PCR was performed using an ABI 7500 fast real-time PCR system (Applied Biosystems, Carlsbad, CA, USA). Universal bacterial primers and TaqMan**®** probes [51] targeted the 331 to 797 *E. coli* numbering region of the 16 S rDNA with forward primer 5′-TCCTACGGGAGGCAGCAGT-3′, reverse primer 5′-GGACTACCAGGGTATCTAATCCTGTT-3′, and the probe, (6-FAM)-5′-CGTATTACCGCGGCTGCTGGCAC-3′-(BHQ1). For this assay, 20 µl qPCR mixtures were prepared including 10 µl of 2× TaqMan**®** Universal PCR master mix with 6-carboxy-X-rhodamine (ROX) passive reference dye (Roche Diagnostics, Indianapolis, IN), 2 µl of 0.4 µg ml^−1^ bovine serum albumin, 0.4 µl of each 10 µM primer, 0.8 µl of 5 µM probe, and 5 µl of DNA template. Thermocycler conditions were 2 minutes at 95°C for initial denaturation and 45 subsequent cycles of 15 seconds at 95°C, 45 seconds at 56°C, and 60 seconds at 72°C. Real-time PCR standard curves of genome quantity versus cycle threshold number for bacteria were developed using known amounts of *Bacillus atrophaeus* (ATCC 49337) genomic DNA. To produce standard curves, five independent dilution series were produced corresponding to 10^1^ to 10^6^ genome copies. For presenting bacterial genome quantities, cycle threshold values were calibrated versus total bacterial genomes. The calibration accounted for the ten rRNA operon copies in *B. atrophaeus* and the average of four rRNA operon copies per genome for all bacteria [26]. To test for PCR inhibition, standard curves for spiked standard *B. atrophaeus* DNA were produced in aerosol filter and sieved floor dust extracts. No inhibitory effects were observed. DNA extracted from filter field blanks was amplified along with the samples, and, if positive, was subtracted from the values obtained for aerosol samples.

### Generation of phylogenetic libraries and data analysis

Phylogenetic library preparation was conducted for sixteen samples and included five indoor occupied PM_10_ filters each from a different sampling day and including one replicate, four occupied duct supply air PM_10_ filters representing each sampling day and a sequencing duplicate, three HVAC filter dust PM_10_ filters each from a separate portion of the filter, three sieved 37-µm floor dust samples each taken on an independent occupied sampling day, and one PM_10_ fraction of resuspended floor dust from one of the 37-µm sieved samples. Table 1 provides additional information on the samples used in phylogenetic analysis. Ribosomal RNA encoding genes were amplified using the 343F and 926R primers [52], [53], which also included sequencing adaptors, keys, and multiplex identifiers. Prior to PCR, primer-dimer formation was screened for each set of primers, barcodes, keys, and sequencing adaptors using OligoAnalyzer 3.1 software. Each PCR reaction was of 25 µl volume and included 1×PCR master mix (Roche Applied Science, Indianapolis, IN), 0.4 µg ml^−1^ of bovine serum albumin, 0.3 µM of each primer, and 3 µl of DNA template. PCR was performed at the following cycling conditions: initial denaturation at 94°C for 5 minutes, and 25 to 35 cycles of 95°C dissociation for 30 seconds, annealing at 47°C for 30 seconds, and extension for 1 minute at 72°C, followed by a final extension at 72°C for 8 minutes. Four PCR reactions were conducted for each sample and amplicons were combined before removing salts and unincorporated primers using a Qiagen MinElute PCR purification kit (Qiagen Inc., Valencia, CA, USA) [54].

Amplicons were visualized on a 1.2% agarose gel and, if necessary, extracted using a Qiagen MinElute gel extraction kit (Qiagen Inc., Valencia, CA, USA). DNA extracts from blank filters were used as negative controls and, as they did not result in amplicons, were not further considered for sequencing preparation. Sequencing was performed at the Yale Center for Genome Analysis. Raw data were subjected to quality control at the machine; keypass, dots, and mixed filters were utilized to assess the quality of the whole read, whereas the quality of read ends was checked by signal intensity and primer filters. Libraries were produced using Roche 454 pyrosequencing and incorporated the GS FLX sequencer and Titanium series chemistry.

Quantitative sequence analysis was performed using tools in the Quantitative Insights Into Microbial Ecology (QIIME) package [55]. Denoising was conducted using Titanium Pyronoise [55], [56], [57]. Sequences were removed from the analysis if they were shorter than 200 base pairs (bp) in length, did not contain the barcode or primer sequence, had any ambiguous nucleotides, produced less than 100 reads per sample, or had a machine-based quality score below 25. Sequences were clustered into OTUs at a minimum identity of 97% and representative sequences were aligned using PyNAST against the greengenes core set from May 2009 [58]. Phylogenetic assignments were made using the naïve Bayesian classifier in the Ribosomal Dataset Project [59].

Fast UniFrac [37] was utilized to produce principal coordinate analyses (PCoA) for comparing the phylogenetic distances between pairs of the 16 samples. Phylotype assignment was made using the RDP classifier and greengenes core set as described above. The resulting PCoA axes were exported and used to produce a graph summarizing the analysis. For PCoA analysis that used previously published datasets including human microflora [31], outdoor air [33], and house dust [20], [27] data, the sequences were assigned to phylotypes by BLASTing against the greengenes database to identify their closest matching sequences [37]. To accomplish this, the greengenes database was formatted into the BLAST database using formatdb and the resulting tree was used to assess the phylogenetic relationships between all examined samples. Each sequence was assigned to its closest BLAST hit in the formatted greengenes database and clustered into phylotypes at 97% sequence similarity for input into Fast UniFrac. Tag information and the unprocessed DNA sequences obtained in this study have been deposited in the MG-RAST archive under accession numbers 40389, 40390, 403991.

### Statistical Analysis

Comparisons between two sets of data were made using an unpaired homoscedastic t-test. Comparisons between more than two values were conducted post-ANOVA analysis via Tukey's test using the Matlab statistical toolbox on Matlab (software version R2010b). Unifrac P-test analysis was performed with the FastUnifrac user interface on the University of Boulder Colorado web interface on http://bmf2.colorado.edu/fastunifrac/index.psp.

## Supporting Information

Figure S1 **HVAC filtration efficiency.** Filtration efficiency was estimated at the HVAC filter—through which indoor return air and outdoor air passes—by placing optical particle counters (size ranges 0.3–0.5 µm, 0.5–1 µm, 1–2.5 µm, 2.5–5 µm, 5–10 µm and >10 µm) before and after the filter. Submicron size particles are inefficiently removed whereas particles bigger than 2.5 µm are removed at 75–90%. The inset is a graphical representation of the air handling unit setup. Dampers were temperature controlled and regulated the relative flow of outdoor air and indoor return air.
(TIF)

Figure S2 **Rarefaction curves for samples of indoor air, ventilation duct supply air, HVAC filter dust, and floor dust.** Curves are based on samples that contained more than 300 sequences to avoid diversity estimate biases. The inset shows the same plot for one floor dust and one indoor air sample, each containing more than 1350 sequences. Error bars represent one standard error using observed species values for independent samples. Chao1 diversity indexes were calculated to be 3720, 1259, 2988, and 637 for floor dust, HVAC filter dust, indoor air, and ventilation duct supply air, respectively.
(TIF)

Figure S3 **Abundance of dominant (A) and rare (B) bacterial phyla from indoor air (Indoor10), ventilation duct supply air (Duct10), HVAC filter dust (HVAC10), and floor dust (Floor10/37).** The dominant phyla represent 93%–98.5% of the sequences recovered. The Cyanobacteria are dominated by chloroplast sequences from plant (*Streptophyta*) material. The number after the samples indicates whether it is a sieved (37, PM_37_) or respirable size fraction sample (10, PM_10_).
(TIF)

Table S1 **Sequencing summary table describing the sample type and listing corresponding sample multiplex identifiers (MIDs).** MIDs were contained on the forward primer. The key adaptor on both primers was TCAG.
(DOC)
