# Supplementary figures and images for: Human Occupancy as a Source of Indoor Airborne Bacteria
Source: PLoS One. 2012 Apr 18;7(4):e34867. doi: 10.1371/journal.pone.0034867 (PMC3329548; doi:10.1371/journal.pone.0034867)

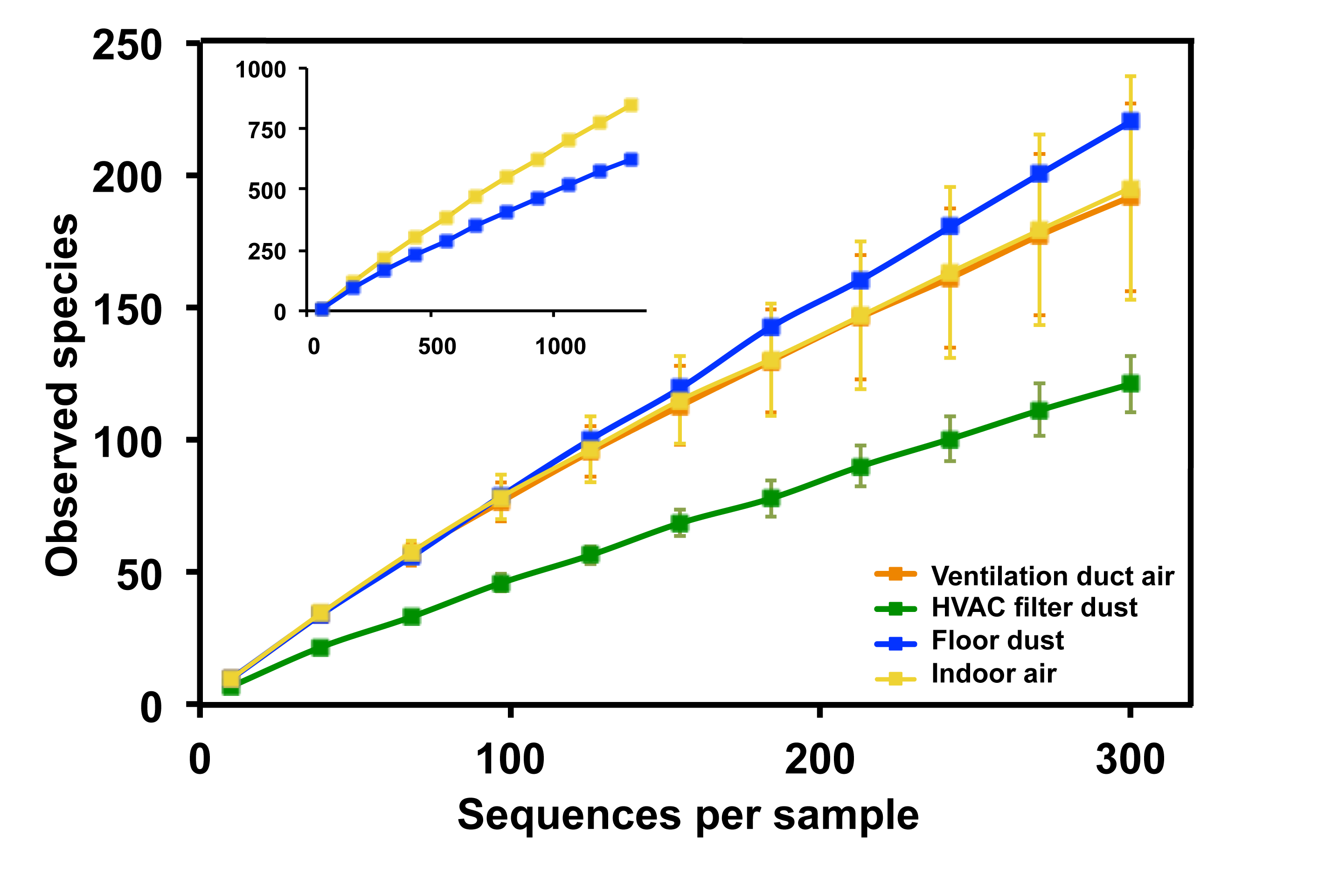

Supplement: Figure S1 — HVAC filtration efficiency. Filtration efficiency was estimated at the HVAC filter—through which indoor return air and outdoor air passes—by placing optical particle counters (size ranges 0.3–0.5 µm, 0.5–1 µm, 1–2.5 µm, 2.5–5 µm, 5–10 µm and >10 µm) before and after the filter. Submicron size particles are inefficiently removed whereas particles bigger than 2.5 µm are removed at 75–90%. The inset is a graphical representation of the air handling unit setup. Dampers were temperature controlled and regulated the relative flow of outdoor air and indoor return air. (TIF) [file pone.0034867.s001.tif]

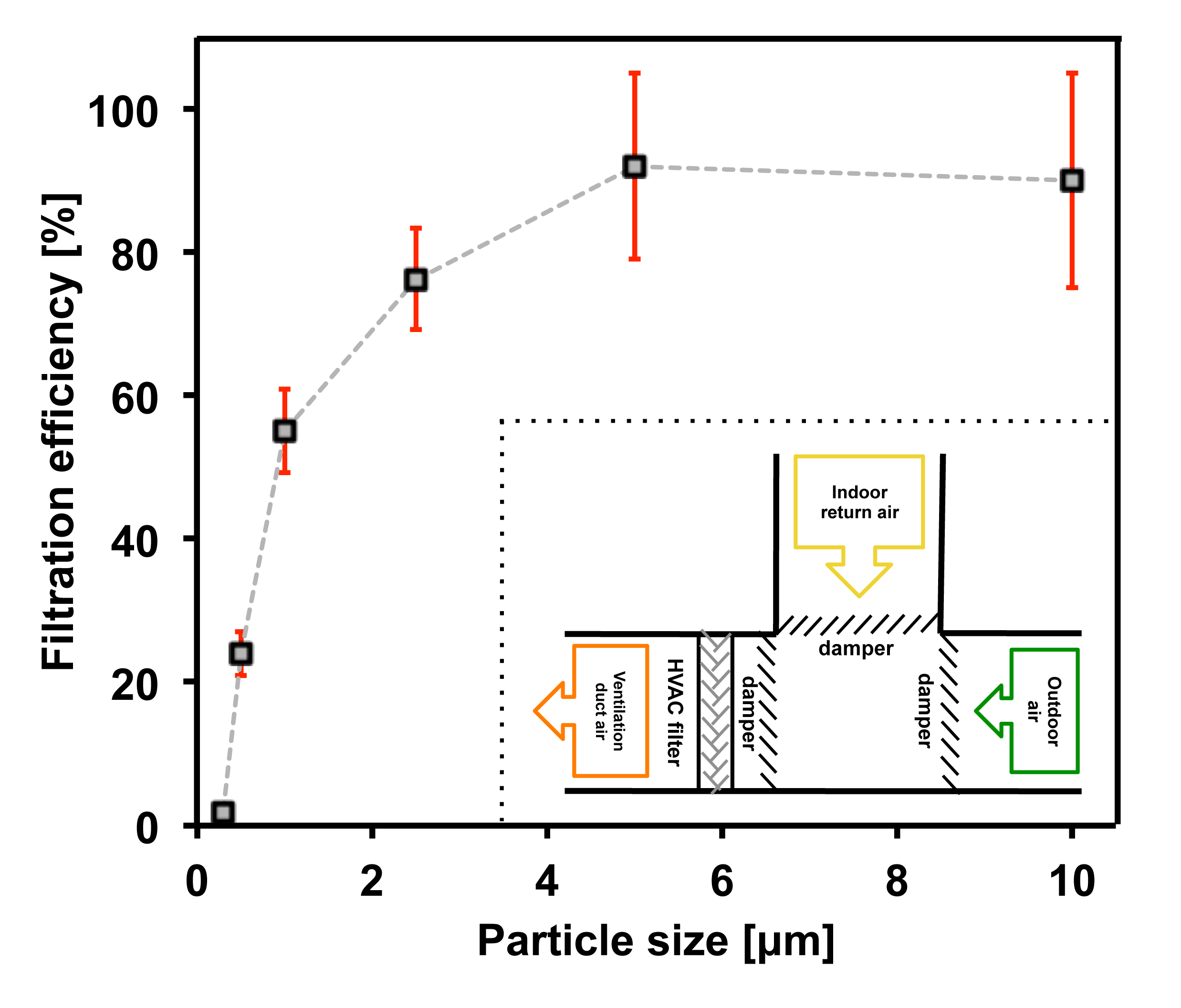

Supplement: Figure S2 — Rarefaction curves for samples of indoor air, ventilation duct supply air, HVAC filter dust, and floor dust. Curves are based on samples that contained more than 300 sequences to avoid diversity estimate biases. The inset shows the same plot for one floor dust and one indoor air sample, each containing more than 1350 sequences. Error bars represent one standard error using observed species values for independent samples. Chao1 diversity indexes were calculated to be 3720, 1259, 2988, and 637 for floor dust, HVAC filter dust, indoor air, and ventilation duct supply air, respectively. (TIF) [file pone.0034867.s002.tif]

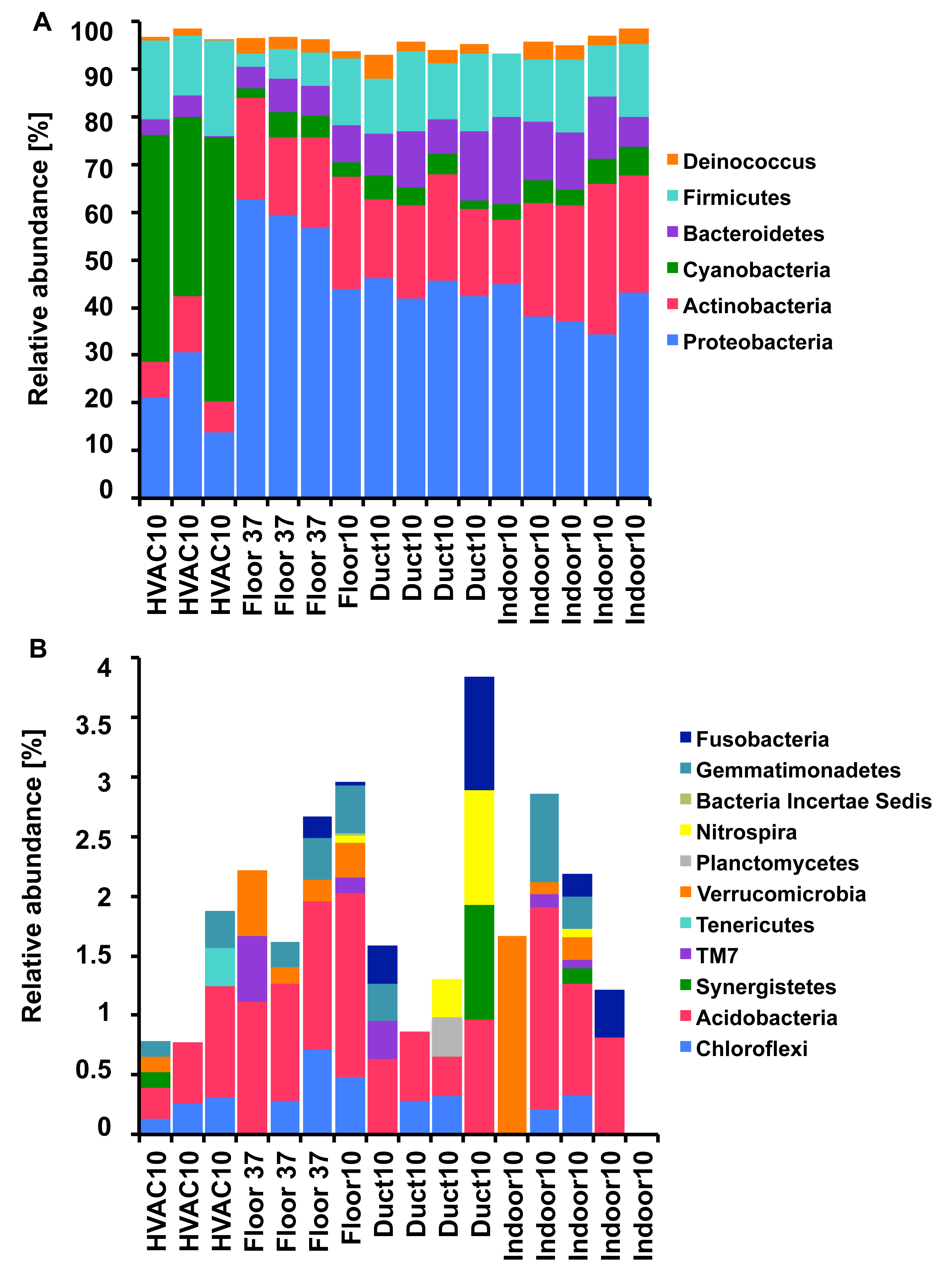

Supplement: Figure S3 — Abundance of dominant (A) and rare (B) bacterial phyla from indoor air (Indoor10), ventilation duct supply air (Duct10), HVAC filter dust (HVAC10), and floor dust (Floor10/37). The dominant phyla represent 93%–98.5% of the sequences recovered. The Cyanobacteria are dominated by chloroplast sequences from plant (Streptophyta) material. The number after the samples indicates whether it is a sieved (37, PM37) or respirable size fraction sample (10, PM10). (TIF) [file pone.0034867.s003.tif]
