# Supplementary material for: Human Occupancy as a Source of Indoor Airborne Bacteria
Source: PLoS One. 2012 Apr 18;7(4):e34867. doi: 10.1371/journal.pone.0034867 (PMC3329548; doi:10.1371/journal.pone.0034867)
Supplement: Table S1 — Sequencing summary table describing the sample type and listing corresponding sample multiplex identifiers (MIDs). MIDs were contained on the forward primer. The key adaptor on both primers was TCAG. (DOC) [file pone.0034867.s004.doc]

| Sample ID | Description | Sampling date | MID |
| --- | --- | --- | --- |
| Indoor air 1 | Indoor air, occupied PM_10_ | November 3^rd^ 2009 | ACGCTCGACA |
| Indoor air 2 | Indoor air, occupied PM_10_ | November 3^rd^ 2009 | AGACGCACTC |
| Indoor air 3 | Indoor air, occupied PM_10_ | November 4^th^ 2009 | AGCACTGTAG |
| Indoor air 4 | Indoor air, occupied PM_10_ | November 4^th^ 2009 | ACGCTCGACA |
| Indoor air 5 | Indoor air, occupied PM_10_ | November 5^th^ 2009 | AGCACTGTAG |
| Ventilation duct supply air 1 | HVAC duct supply air occupied PM_10_ | November 3^rd^ 2009 | TGTACTACTC |
| Ventilation duct supply air 2 | HVAC duct supply air occupied PM_10_ | November 4^th^ 2009 | CGTAGACTAG |
| Ventilation duct supply air 3 | HVAC duct supply air occupied PM_10_ | November 5^th^ 2009 | TAGAGACGAG |
| Ventilation duct supply air 4 | HVAC duct supply air occupied PM_10_ (sieved and resuspended) | November 5^th^ 2009 | CGTAGACTAG |
| Floor dust 1 | PM_37_ (sieved) | November 3^rd^ 2009 | TCTGACGTCA |
| Floor dust 2 | PM_37_ (sieved) | November 4^th^ 2009 | TGTCGTCGCA |
| Floor dust 3 | PM_37_ (sieved) | November 5^th^ 2009 | TCTGACGTCA |
| Floor dust 4 | PM_10_ (sieved and resuspended) | November 3^rd^ 2009 | AGTATACATA |
| HVAC filter dust 1 | PM_10_ (sieved and resuspended) | February 28^th^ 2010 | TATATATACA |
| HVAC filter dust 2 | PM_10_ (sieved and resuspended) | February 28^th^ 2010 | TACGTCATCA |
| HVAC filter dust 3 | PM_10_ (sieved and resuspended) | February 28^th^ 2010 | TAGTCGCATA |
